# Multimodal MRI of white matter development and selective motor control in preterm infants

**DOI:** 10.1016/j.nicl.2026.103991

**Published:** 2026-04-02

**Authors:** Alexander Drobyshevsky, Vasiliy Yarnykh, Theresa Sukal Moulton, Andrea C. Pardo, Raye Ann deRegnier, Colleen Peyton

**Affiliations:** a Department of Pediatrics, Endeavor Health, Evanston, IL, USA; b Department of Radiology, University of Washington, Seattle, WA, USA; c Department of Pediatrics, Northwestern University, Feinberg School of Medicine, USA; d Lurie Children’s Hospital, Northwestern University, USA; e Department of Physical Therapy and Human Movement Sciences, Northwestern University, Chicago, IL, USA

## Abstract

**Background:**

Selective motor control (SMC), the capacity to isolate joint movements, emerges in infancy and is a hallmark of typical development (TD). In infants with cerebral palsy (CP), SMC predicts future motor abilities. Although SMC is thought to reflect increasing cortical influence, its neural substrates remain poorly characterized. This study employed multimodal MRI to investigate the relationship between corticospinal tract (CST) microstructure and SMC in very preterm infants at high risk of CP.

**Methods:**

15 very preterm infants, born before 32 weeks’ gestation with birthweight < 1500 g, underwent one or two MRI exams (total 20) between 3- and 21-week corrected age. Imaging included macromolecular proton fraction (MPF) mapping to quantify myelin, diffusion tensor imaging (DTI) to assess fractional anisotropy (FA), and g-ratio estimation. SMC was evaluated at the time of MRI. Regions of interest included the posterior limb of the internal capsule (PLIC), rubrospinal tract, and corpus callosum.

**Results:**

Of the 15 infants studied, at the Bayley-4 assessment at 2 years, one was later diagnosed with spastic CP, one infant exhibited early motor delay but walked independently by age 2, and the remaining 13 had typical motor development. In infants with typical development, SMC scores increased with age and correlated with MPF in the PLIC (R^2^ = 0.81). The rate of MPF increase in the CST outpaced that of the corpus callosum, 3.58% vs. 1.11% per week. In the infant with CP, a left hemisphere injury was associated with reduced MPF in the left CST and lower contralateral SMC, whereas rubrospinal MPF was elevated on the ipsilesional side.

**Conclusion:**

Early CST myelination parallels the emergence of SMC. Quantitative myelin imaging, particularly MPF, may serve as a sensitive structural biomarker of motor system maturation and emerging motor control, enabling early detection and intervention in CP.

## Highlights

• Corticospinal tract myelination closely tracks the emergence of selective motor control in very preterm infants.
• Myelin-sensitive MRI provides a stronger structure–function link than diffusion metrics alone.
• Early deviations in cortico-/rubrospinal myelination identify atypical motor pathway development relevant to cerebral palsy.

## 1 Introduction

Cerebral palsy (CP), the most common cause of lifelong motor disability in childhood, with a prevalence of 3.2 per 1000 children in the USA (McGuire et al., 2019), most often arises from early brain injury disrupting development of the motor system (Reddihough and Collins, 2003). Early white and gray matter injury detected on MRI at term equivalent age (TEA) has been shown to predict adverse neurological outcome at 2 years of age (Counsell et al., 2008, Pagnozzi et al., 2025, Woodward et al., 2006). However, combining structural MRI measures with early functional assessment of motor function at 3–4 months corrected gestational age (CGA) improves predictive accuracy with TEA MRI alone (Setanen et al., 2014, Wang et al., 2021).

One of the earliest and most functionally significant consequences of early brain injury, particularly affecting the corticospinal tract (CST), is impaired selective motor control (SMC), defined as the ability to isolate individual joint movements, and is essential for both gross (Fowler et al., 2009) and fine motor function (Sukal-Moulton et al., 2018). The CST enables SMC by precisely timing motor commands from the sensorimotor cortex to spinal motor neurons, thereby coordinating individual movements across multiple joints. SMC impairment is a defining feature of CP and a robust early predictor of motor outcome (Peyton et al., 2024a). The recently validated Baby Observational Selective Control AppRaisal (BabyOSCAR) provides a standardized, quantitative measure of early SMC and predicts spastic CP diagnosis by age two with 98% sensitivity and 100% specificity (Barbosa et al., 2024, Peyton et al., 2024a, Peyton et al., 2024b, Sukal-Moulton et al., 2024a). Given that the early infancy period between 0 and 6 months of CGA represents the critical period when SMC emerges (Peyton et al., 2025a), studying this process in very preterm infants, who have the highest probability of CP and are associated with atypical SMC development, has both scientific and clinical significance. Understanding how motor circuit development in this population matures could enable earlier identification and targeted intervention for CP.

Insights from animal studies indicate that the maturation of the CST, which involves axonal pruning, progressive myelination, and refinement of synaptic connections, is essential for the development of complex, coordinated motor behaviors (Chakrabarty et al., 2009, Martin, 2005). Among these developmental processes, myelination plays a central role in determining white matter functional capacity. By regulating conduction velocity, reducing temporal delays, and improving the synchrony and reliability of signal transmission, myelination enables efficient integration of distributed motor networks (Pajevic et al., 2014). Disruption of these processes during early development in preterm infants or following perinatal brain injury can impair CST maturation and alter the balance between descending cortical control and spinal excitability (Drobyshevsky et al., 2007, Synowiec et al., 2018). Such disturbances contribute to abnormal motor synergies and diminished selective motor control characteristic of cerebral palsy (Vuong et al., 2021). Thus, the degree and pattern of myelination within the developing CST not only reflect white matter integrity but also critically determine the brain’s capacity for precise and independent motor control.

In humans, our understanding of CST development has relied primarily on diffusion weighted MRI, which provides scalar metrics sensitive to axonal coherence and organization (Le Bihan et al., 2001). However, diffusion-based measures are non-specific and do not directly reflect myelin content. Recent advances in quantitative MRI allow more specific characterization of white matter microstructure. Macromolecular proton fraction (MPF) mapping, derived from quantitative magnetization transfer imaging, provides a reproducible and biophysically grounded estimate of myelin content in vivo (Yarnykh et al., xxxx, Yarnykh, 2016b). When combined with DTI-based axonal volume fraction, these data enable estimation of the g-ratio, an integrated index of axon–myelin coupling that reflects the efficiency of neural conduction (Stikov et al., 2015).

Despite these advances, few studies have applied multimodal quantitative MRI to link white matter development with emerging motor function during early infancy. This gap limits our ability to identify structural–functional biomarkers that reflect the maturation of corticospinal regulation underlying SMC. This study employs complementary quantitative MRI techniques, DTI, MPF, and derived g-ratio estimates, to characterize CST development in preterm infants during the first five postnatal months. We relate these imaging findings to performance on BabyOSCAR (Sukal‐Moulton et al., 2024b). By linking structure and function in early infancy, this work aims to identify potential imaging biomarkers of emerging motor control and support earlier, more targeted intervention strategies, particularly in medically complex infants, where health issues may delay behavioral assessment.

## 2 Methods

### 2.1 Participants

Fifteen preterm infants (<32 weeks' gestational age; <1500 g birthweight) were prospectively enrolled between June 2022 and March 2023 as part of a larger study on SMC. None had congenital malformations or known genetic syndromes. All underwent MRI between 3 and 21 weeks' corrected gestational age (CGA) under a research protocol approved by the local Institutional Review Board. Five infants underwent two serial examinations, yielding a total of 20 MRI datasets. Standardized developmental assessments using the Bayley Scales of Infant and Toddler Development, 4th edition (Bayley, 2006), were completed at 18–24 months CGA as part of routine clinical care. Typical motor development was determined if the Bayley Motor Composite score was above 85 (within 1SD of the mean).

Selective motor control assessment. Infants were scored every 2–4 weeks from enrollment to 5 months CGA using video recordings and the Baby Observational Selective Control Appraisal (BabyOSCAR) tool (Sukal-Moulton et al., 2024a), which evaluates joint isolation, synergies, and mirror movements. Two trained raters independently scored each joint based on a 1-minute video segment. The total combined score ranges from 0 to 32.

### 2.2 MRI imaging protocol

Subjects were scanned at Lurie Children’s Hospital using a Siemens 3 T system, with a parent present. Before scanning, infants were fed and swaddled; no sedatives or narcotics were administered. Imaging was performed using a 24-channel head coil array. Clinical reading of MRI images and injury grading was performed by pediatric neuroradiologists. The MRI images were processed by one of the authors (A.D.), who has more than 20 years of experience in neonatal imaging.

### 2.3 Macromolecular proton fraction (MPF) mapping

MPF data were acquired using a sagittal 3D spoiled gradient echo protocol with three contrasts (T1, PD, and MT), each with specified TR/TE/flip angles (e.g., 16/4.6/18 for T1). The acquisition voxel size was 1.5 mm^3^ isotropic, and the field of view was 160 mm^2^. Total scan time was ∼13 min. Reconstruction of MPF maps was performed using a two-pool model implemented in custom C++ software, incorporating a synthetic reference for the single-point algorithm (macromolecularmri.org) (Yarnykh, 2016a).

### 2.4 Diffusion tensor imaging (DTI)

was conducted with a single-shot EPI sequence aligned to the AC–PC line (1.92 × 1.9 mm in-plane resolution, 68 slices, 1.9 mm thickness, FOV 180 × 140 mm, matrix 94 × 94, TR/TE = 8000/74 ms, NEX = 1, SENSE = 2). The protocol included 64 diffusion directions (b = 1000 s/mm^2^)^2^ plus a reverse-phase b = 0 image for distortion correction using FSL’s topup tool. Acquisition time was 9 min 21 sec. FA, mean, and directional diffusivity maps were computed using FSL’s diffusion toolbox (Jenkinson et al., 2012).

### 2.5 Aggregate g-ratio mapping

The g-ratio, defined as the inner-to-outer diameter of myelinated axons, was estimated from single-shell DTI (Cortina et al., 2022) using:

g-ratio = √1/(1 + MVF/AVF) (Stikov et al., 2015),

where MVF is myelin volume fraction, and AVF is axonal volume fraction. MVF was derived from MPF using geometric scaling (Jung et al. (Jung et al., 2018)), and AVF was calculated as:

AVF = (1 − MVF) × ICVF,

with ICVF derived from NODDI-DTI (Edwards et al., 2017) using a single-shell diffusion model and an in-house MATLAB script.

### 2.6 Region of interest (ROI) determination

**Fig. 1:**
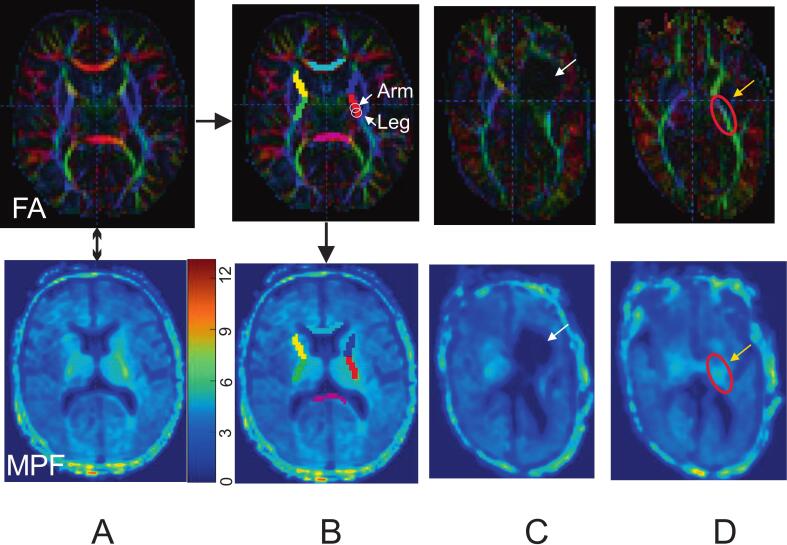
Multimodal assessment of major white matter tracts in a typically developing infant at 17 weeks CGA (A, B) and in an infant with CP at 9 weeks CGA (C, D). A. FA and MPF maps were spatially co-registered. The color bar (A, bottom row) shows the macromolecular proton fraction as a percentage. B. Regions of interest (ROIs) were manually outlined bilaterally on color directionally encoded FA maps (top row) and transferred to the co-registered MPF maps (bottom row). Portions of PLIC were also delineated for CST segments that mainly carried motor fibers controlling the arms and legs (B, top row). C. Color directionally encoded FA maps depict an infant with CP with periventricular leukomalacia at 9 weeks. The white arrow on the FA map points to the lesion location and injury to the PLIC. ROIs were placed on a neighboring slice where the CST tract was visibly preserved in the same infant with CP (D).

MPF maps were co-registered to DTI scans using FSL’s nonlinear registration tool (Jenkinson et al., 2012). Regions of interest (ROIs) were delineated bilaterally in the posterior limb of internal capsule (PLIC) and corpus callosum (CC) using FA color maps in ITK-SNAP (https://www.itksnap.org/), with placement standardized to the splenium level (Fig. 1). To target specific descending motor pathways, PLIC was divided into four equal segments, with ROIs placed on the third and fourth posterior segments, representing arm and leg projections (Pan et al., 2012) (Fig. 1B, top). Rubrospinal ROIs were defined around the red nucleus across three axial slices, guided by diffusion features and established anatomical references (Owen et al., 2017, Yang et al., 2011). To reflect the relative progression of maturation, extracted FA and MPF values were normalized by subtracting the starting values in fetuses, dividing by the developmental range (from fetal to maximum adult values), and expressed as percent change. The developmental ranges of FA in PLIC (0.2–0.68), CC (0.2–0.85), and MPF in PLIC (2%.5–13.4%), CC (2.5%-13.2%) were based on literature data from fetuses and adults (Korostyshevskaya et al., 2019, Oishi et al., 2011).

For cortical analysis, MPF maps were registered to the University of North Carolina infant template (Shi et al., 2011) using T1-weighted scans and affine registration, followed by nonlinear registration in ANTs (Avants et al., 2011). Cortical ROIs were then transferred to MPF maps, and average values were reported.

### 2.7 Statistical analysis

A linear mixed-effects model was used to assess the association among corrected gestational age (CGA), BabyOSCAR scores, and MRI indices in the typically developing group. Because some infants contributed repeated measurements, subject ID was included as a random intercept to account for within-subject dependence. Because there were few repeated studies, random slope estimation was not included in the model. The model was fit in MATLAB (fitlme) using maximum likelihood estimation, and fixed effects were evaluated using Wald t-tests and Type III F-tests. Model assumptions were checked using standard residual diagnostics. 95% prediction intervals were constructed using the model's residual standard deviation. Data from infants with atypical motor development were not used to fit the model. These data were instead tested to assess whether they fell outside the prediction interval of the typical development population mean trajectory. Sex and the presence of bronchopulmonary dysplasia were initially included as fixed effects in the mixed-effects models. As these factors were not statistically significant, they were omitted from the final models.

Differences in age-related MPF and FA trajectories between regions in the typically developing group were assessed using linear mixed-effects models with fixed effects for CGA, region, and their interaction, and random intercepts and CGA slopes for subject. A significant CGA × region interaction was interpreted as evidence of differing developmental slopes.

The association between BabyOSCAR scores and MRI parameters was assessed using Pearson correlation. p-values < 0.05 were considered significant.

## 3 Results

**Fig. 2:**
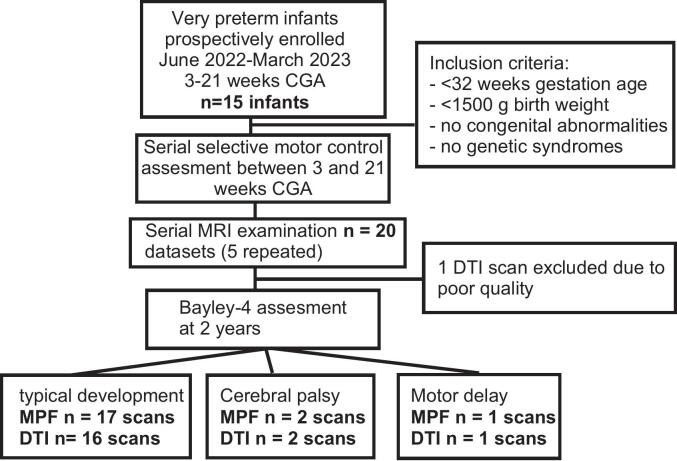
Patient flow diagram according to the STROBE guidelines.

Fig. 2 presents a flow diagram of patient selection and examinations performed. Of 20 MRI studies, 1 DTI scan was excluded due to motion and poor quality, leaving 19 DTI scans and 20 MPF maps for analysis. Patients' demographics are in Table 1. Most infants (n = 13, 87%) exhibited typical motor development on the Bayley-4 (Bayley, 1993) at ≥18 months corrected age. One infant had motor delay (Bayley Motor Composite <85), and one with spastic CP (history of unilateral IVH grade IV, PVL, and VP shunt) was classified as GMFCS IV at 4 years with bilateral lower and right upper extremity involvement. These two cases were excluded from age-based regressions and plotted separately. Four typically developing children and the child with CP had two MRI examinations.

**Table 1:** Demographic and clinical characteristics of the study participants. The data are expressed as median value (interquartile range), or number (percentage of sample).

Number of participants	15
Gestational age (weeks)	29 (28, 30)
Range of gestational age, weeks (min, max)	24, 31
Birthweight (g)	1080 (945, 1285)
Female	6 (40)
Race	
Asian	3 (20)
Black or African American	1 (7)
White	9 (60)
Unknown	2 (13)
Ethnicity	
Hispanic or Latino	2 (13)
Not Hispanic or Latino	12 (80)
Unknown	1 (7)
Intraventricular Hemorrhage	2 (13)^a^
Bronchopulmonary dysplasia^b^	10 (67)
Treated retinopathy of prematurity^c^	3 (20)
Necrotizing enterocolitis^d^	0 (0)

a One subject with bilateral grade II, one subject with grade unilateral grade IV.

b Required supplemental oxygen ≥35 weeks gestation.

c Treated with Avastin and/or laser therapy.

d Treated with drain or laparoscopy.

### 3.1 Indexes of the selective motor control increased with age, and were lower in impaired limbs of the individual with cerebral palsy

**Fig. 3:**
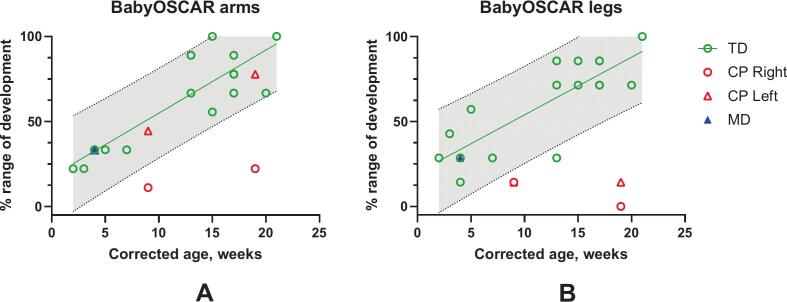
Selective motor control increases in the arms (A) and legs (B) are observed in typically developing (TD) children and in the infant with cerebral palsy. The population regression line and 95% prediction intervals are shown for the normalized babyOSCAR score of arms and legs. Data for typically developing infants are averaged from the left and right sides. Data for the infant with cerebral palsy (CP) are presented separately for the right (contralateral to the lesion) and left (ipsilateral to the lesion) sides. Data for the infant with motor delay are labeled as MD.

Indices of SMC for arms and legs in infants with typical motor development gradually increased between 1 and 5 months CGA, as shown in Fig. 3, and approached a maximum score of 7 for legs and 9 for arms. For comparison of rate changes in the arms and legs, the babyOSCAR scores were normalized to the respective ranges for 1–5 months of CGA and expressed as percentages. Populational slopes were similar for arms (3.45%/week, t_15_ = 10.07, p < 0.001) and legs (4.26%/week, t_15_ = 11.43, p < 0.0001). In the infant with CP, SMC scores were below the 95% prediction interval for age, except for the ipsilesional left arm. In contrast, the infant with motor delay showed scores within the typical range.

### 3.2 Rapid corticospinal tract myelination, detectable with MPF, outpaces callosal development in early infancy

**Fig. 4:**
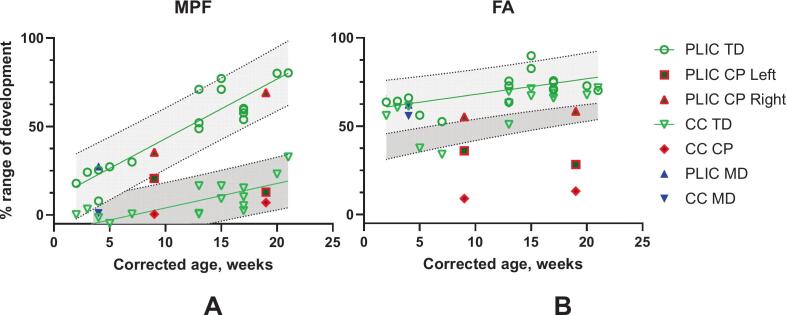
Developmental changes in MPF (A) and FA (B) in the posterior limb of the internal capsule (PLIC) and the corpus callosum (CC) in typically developing (TD) infants and infants with cerebral palsy (CP) and motor delay (MD). The population regression line and 95% prediction intervals are based on TD infants' data, whose values reflect the averages of left and right. CP infant data are presented separately for the right (contralateral to the lesion) and left (ipsilateral) sides and are highlighted in red. MD infant data are shown as left- and right-sided averages for each region. (For interpretation of the references to colour in this figure legend, the reader is referred to the web version of this article.)

In infants with typical motor development, mean populational MPF in the PLIC increased from 24% to 80% of adult values between 2 and 21 CGA weeks. (Fig. 4A), with a slope of 3.58% per week (t_14_ = 12.38, p < 0.001). In contrast, MPF in the corpus callosum rose more slowly (0.5% to 32%; slope = 1.30% per week, t_15_ = 3.90, p < 0.001). The PLIC myelinated significantly faster than the callosum (F_1_,_1_ = 37.92, p < 0.0001). In the infant with CP, the MPF in the lesioned PLIC was below the 95% confidence interval, whereas the non-lesioned PLIC was within the typical range.

FA showed smaller, more uniform increases: 52–89% in the PLIC and 37–71% in the CC (Fig. 4B), with regression slopes of 0.95% per week t_14_ = 3.16, p = 0.06 and 1.11% per week (t_14_ = 3.99y, p = 0.001. FA slopes did not differ between regions (F_1,1_ = 0.749, p = 0.3941). In the infant with CP, FA in the lesioned PLIC and CC were both below the 95% prediction range of typical development, whereas the non-lesioned PLIC was within the typical range. FA and MPF values in the infant with motor delay fell within typical limits.

### 3.3 Myelination in the corticospinal tract closely parallels the emergence of selective motor control

**Fig. 5:**
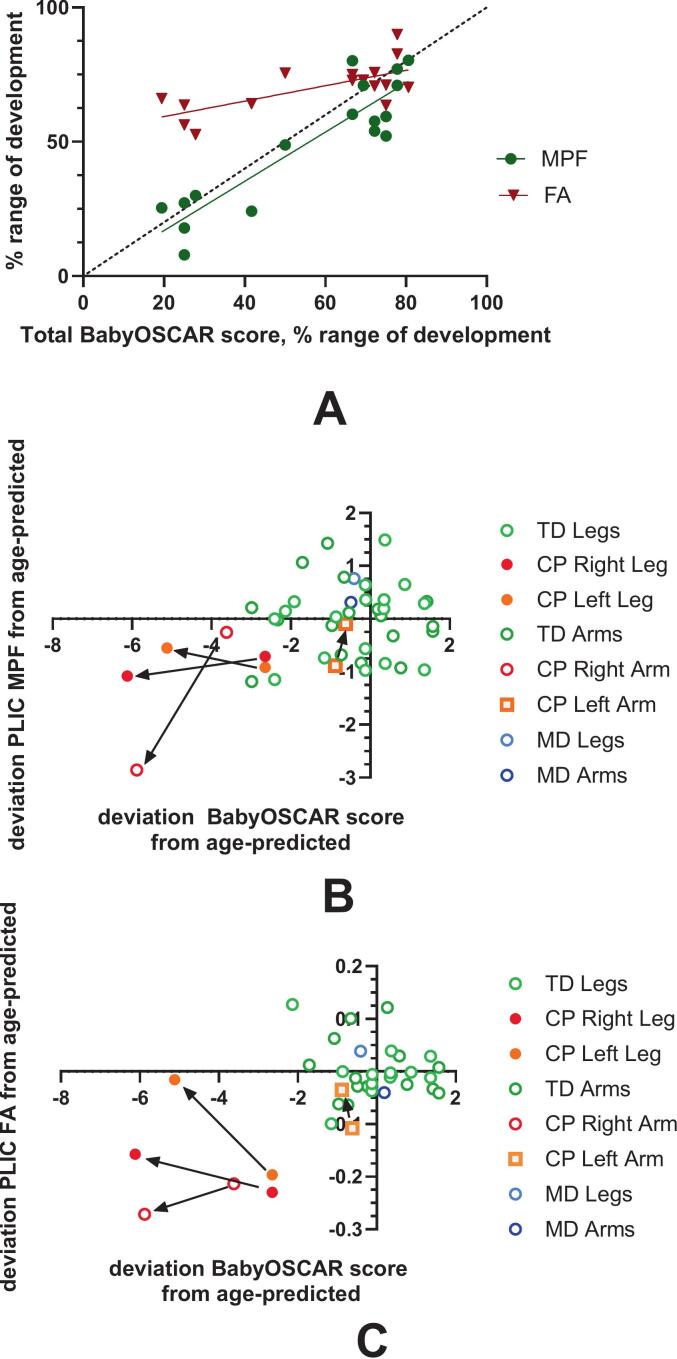
Relationship between selective motor control and corticospinal tract development. Normalized total BabyOSCAR scores plotted against normalized MPF and FA values in the posterior limb of the internal capsule (PLIC) for typically developing (TD) infants. Scores represent bilateral averages for arms and legs. (B) MPF and (C) FA deviations from age-predicted values are plotted against BabyOSCAR scores in the infant with cerebral palsy (CP), shown separately for each limb. Imaging values represent the PLIC contralateral to the tested limb (i.e., corresponding to the corticospinal tract serving that limb). The infant with motor delay (MD), who did not develop CP, is shown with bilateral averages for both arms and legs. Arrows in B and C indicate the longitudinal change in the CP infant, highlighting increasing deviation from typical development over time. TD Arms, TD Legs = bilateral averages in typically developing infants; CP Right Arm, CP Left Arm, CP Right Leg, CP Left Leg = individual limb scores and contralateral imaging values in the infant with CP. MD Legs, MD Arms = bilateral averages in the infant with motor delay.

Normalized total BabyOSCAR scores increased with age in typically developing infants and aligned more closely with MPF than FA values in the PLIC (Fig. 5A). BabyOSCAR scores were strongly associated with MPF (Pearson r = 0.91, R^2^ = 0.81, p < 0.0001) with close alignment to the identity line. Association of babyOSCAR and FA were moderate FA (r = 0.69, R^2^ = 0.47, p = 0.003). To depict temporal dynamics of the babyOSCAR and structural MRI indexes in infants with abnormal development, deviations from actual scores and those predicted for the corresponding age from the mixed models for typically developing infants (Fig. 4) are plotted for babyOSCAR, MPF and FA (Fig. 5 B,C). For the infant with CP, behavioral and MRI values fell within the lower left quadrant of Fig. 5B and C, indicating delays in both functional and structural CST development. These deviations increased over time (9–19 weeks CGA) relative to the first measurement, particularly in the arm contralateral to the PVL lesion, as reflected in both BabyOSCAR and PLIC MPF/FA values. The infant with motor delay, however, remained within the 95% confidence range for typically developing infants across all measures.

### 3.4 G-ratio development in projection and commissural fibers

**Fig. 6:**
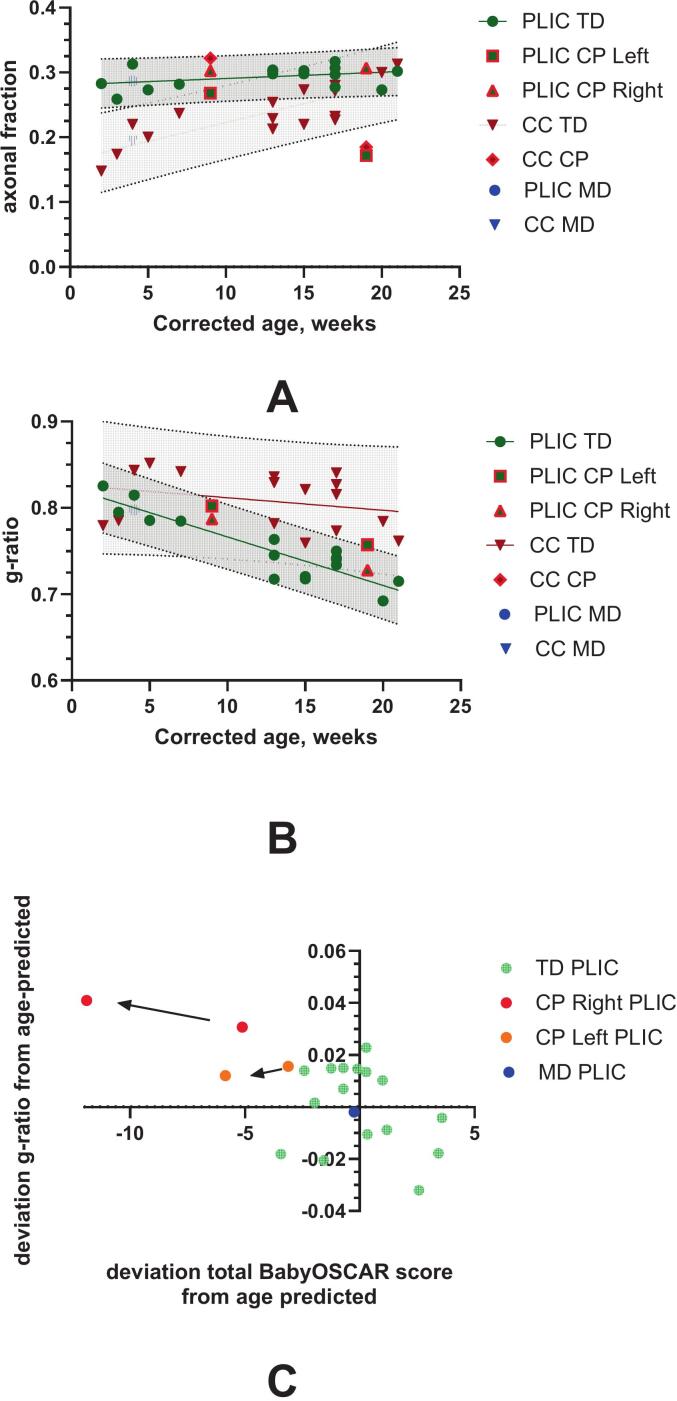
Developmental changes in axonal fraction (A) and g-ratio (B) in the posterior limb of the internal capsule (PLIC) and the corpus callosum (CC) in typically developing infants, infants with motor delay (MD), and infants with cerebral palsy (CP). The linear regression line and 95% confidence intervals are shown. Data for typically developing infants are presented as averages from both the left and right sides. Data for the infant with CP are shown separately for the contralesional and ipsilesional sides, marked with a red border. C. Deviations of the actual g-ratio from the predicted values using linear regression versus age between 3 and 21 weeks of CGA for the typically developing infants and the infant with CP. The BabyOSCAR score is plotted for the limb contralateral to the internal capsule side on MRI. Arrows indicate the transition between the first and second MRI study at 9 and 19 weeks of CGA in the infant with CP. (For interpretation of the references to colour in this figure legend, the reader is referred to the web version of this article.)

In typically developing infants, the axonal fraction (measured with NODDI-DTI) increased gradually in the PLIC from 3 to 21 weeks CGA (Fig. 6A). The rate of increase in the PLIC was 0.96E-3 per week (t_14_ = 1.60, p = 0.13, R^2^ = 0.13), not significantly different from zero, while in the CC, it was 5.8E-3 per week (t_14_ = 9.7, R^2^ = 0.68, p < 0.0001). The infant with CP showed a lower axonal fraction in the PLIC, falling below the 95% confidence interval. The higher slope in the CC (F_1,1_ = 17.81, p = 0.0002) indicates ongoing axonal growth in commissural tracts during this time, compared to more mature axonal development in projection tracts like the PLIC. The infant with motor delay had values within the typical range.

The g-ratio decreased more sharply in the PLIC than in the CC (Fig. 6B), with a slope difference of F_1,1_ = 17.82, p = 0.0002. The PLIC slope was −5.6E-3 per week (t_14_ = -18.2, p < 0.0001, R^2^ = 0.82), whereas the CC slope was −1.45E-3 per week (t_14_ = -1.16, p = 0.26, R^2^ = 0.078), which was not significantly different from zero. In the infant with CP, the ipsilesional PLIC was elevated at 9 and 19 weeks, exceeding the 95% confidence interval, while contralesional values remained within the normal range. This deviation mirrored the worsening of the BabyOSCAR score over time (Fig. 6C).

### 3.5 Rubrospinal myelination increases with age and is elevated in CP

**Fig. 7:**
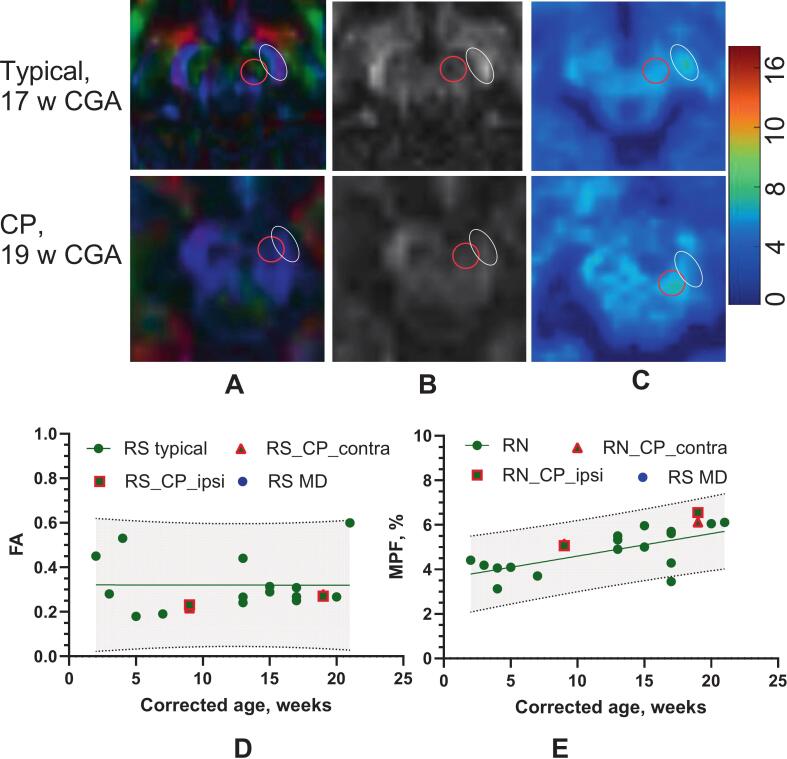
Rubrospinal tract between 2 and 21 CGA. The rubrospinal tract was outlined by placing ROIs on the red nucleus in the mesencephalon and adjacent slices, using a color-encoded FA map (A, red circle), and then transferred to the FA map (B) and MPF map (C). The color bar shows the macromolecule proton fraction mapping as a percentage. The top row depicts an infant with typical motor development at 17 weeks corrected gestational age (CGA), while the second row shows an infant with cerebral palsy (CP) at 19 weeks CGA. Note the decreased anisotropy in the ipsilesional cerebral peduncle (white oval) and increased MPF in the rubrospinal tract (red circle). D, E. − Developmental changes in FA (D) and MPF (E) for typically developing infants and an infant with cerebral palsy. The linear regression line with 95% confidence intervals is shown. Data for typically developing infants are averaged from both sides. Data for the infant with cerebral palsy (CP) are shown separately for the right (contralateral to the lesion) and left (ipsilateral to the lesion) sides, marked in red. MD – data for the infant with motor delay. (For interpretation of the references to colour in this figure legend, the reader is referred to the web version of this article.)

MPF values in the rubrospinal tract increased with age between 3 and 21 weeks CGA in typically developing infants (0.11%/week, t_15_ = 4.49, p < 0.0001, Fig. 7A), whereas FA showed no apparent change during this period (p = 0.99, Fig. 7B). In the infant with CP, rubrospinal MPF was higher than in typically developing infants, possibly indicating compensatory reliance on bulbospinal pathways following CST injury.

### 3.6 Myelination gradually increases between 3 and 21 weeks in the cerebral cortex

**Fig. 8:**
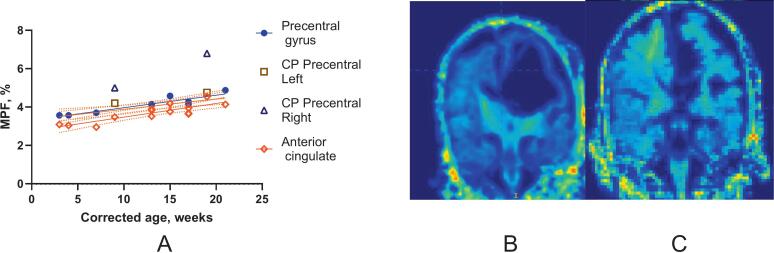
Cortical myelination between 3 and 21 weeks CGA. A. MPF changes in motor and non-motor cortex with age in typically developing infants and infants with CP, ipsilateral and contralateral to the lesion. MPF maps at 9 (B) and 19 (C) weeks in an infant with CP on a coronal section through the sensorimotor cortex. The arrow indicates increased MPF in the perirolandic area.

Cortical myelination increased with age between 1 and 5 months across all regions, including the precentral and anterior cingulate areas (Fig. 8). MPF changes were similar between motor-related and other cortical regions, with no distinct pattern in motor-related cortex linked to emerging SMC. Notably, in the infant with CP, cortical myelination was elevated contralateral to the lesion, as shown in MPF coronal sections at two time points (Fig. 8, triangle markers; Fig. 8B, C).

## 4 Discussion

This study demonstrates that early white matter microstructural features of the corticospinal tract, particularly the MPF and g-ratio estimates, are significantly associated with the emergence of SMC in preterm infants. To our knowledge, this is the first study to apply MPF and g-ratio imaging to characterize CST development in relation to motor behavior in human infants. These myelin-sensitive measures offer enhanced specificity over traditional scalar DTI metrics, which are confounded by multiple microstructural factors. These findings extend prior work linking CST maturation to motor function (Eyre et al., 2007) by providing quantitative evidence of structural–functional coupling within the first 5 months of life.

### 4.1 Differential maturation of white matter tracts

Myelination, as quantified by MPF, showed a rapid postnatal increase in the CST, particularly within the PLIC, during the first five months after term. In contrast, the corpus callosum, still in early stages of myelination, exhibited minimal MPF change (0.5–32% of adult values), consistent with its known protracted development (Luders et al., 2010). FA, which reflects white matter organization, but not myelin directly (Friedrich et al., 2020), followed a different profile: it began at a higher baseline, increased less steeply, and showed similar trajectories in PLIC and in the corpus callosum. These findings suggest that while structural coherence, indicated by FA, is established early, MPF captures a more dynamic and functionally relevant phase of CST maturation during early infancy. This interpretation aligns with prior evidence supporting MPF’s sensitivity to regionally specific, activity-dependent developmental trajectories in both gray and white matter (Drobyshevsky et al., 2023).

### 4.2 Myelination and Selective motor control

A key finding of this study is the high correlation between the rapid CST myelination (by MPF in the PLIC) and SMC emergence. Normalized Total BabyOSCAR scores increased proportionally and aligned more closely with MPF than FA (Fig. 5A), supporting our hypothesis that the development of SMC reflects increasing descending functional drive from the maturing corticospinal system. Fast, efficient conduction along these myelinated fibers is essential for the precise timing required for isolated joint control. Because oligodendrocytes preferentially myelinate active axons (Wake et al., 2015), MPF may also reflect functional engagement. These findings highlight MPF as a promising marker of circuit maturation underlying early motor control.

### 4.3 Insights from the cerebral palsy case study

The single case of the infant with spastic CP offered preliminary insight into atypical development. This infant, with a history of IVH grade IV and PVL, showed significant differences in both behavioral (BabyOSCAR scores) and structural/functional MRI indices (MPF, FA, g-ratio) in the ipsilesional PLIC. The age-related increase during 9–19 weeks CGA mirrors prior behavioral findings (Peyton et al., 2025b) of progressive corticospinal disruption.

As expected, the right arm (contralateral to the PVL lesion) was more affected, reflected in lower BabyOSCAR scores and MPF/FA changes in the corresponding CST. Bilateral lower extremity involvement was also observed, possibly due to early bilateral CST organization (Eyre et al., 2001). BabyOSCAR scores indicated bilateral lower-limb involvement not fully anticipated by imaging alone. This highlights the value of pairing pathway-specific imaging with behavioral assessment to anticipate later motor impairments and tailor early interventions.

G-ratio analysis revealed a greater developmental decrease in the PLIC than in the corpus callosum (Fig. 6B), approaching adult-like values of ∼0.7 (Cercignani et al., 2017, Mohammadi and Callaghan, 2021). In the infant with CP, the ipsilateral PLIC g-ratio remained above the 95% CI at both timepoints, indicating inefficient myelination that paralleled lower BabyOSCAR scores (Fig. 6C). The contralesional PLIC g-ratio remained within normal limits, reinforcing the lesion-specific effects.

In the corpus callosum of the infant with CP, MPF was within the typical range. However, FA was notably reduced (Fig. 4), suggesting a different impact of perinatal injury on commissural vs. projection tracts or compensatory myelination not captured by FA. Similarly, although cortical myelination generally increased with age (Fig. 8), the infant with CP showed elevated myelination contralateral to the lesion (Fig. 8B, C), possibly reflecting compensatory reorganization.

Finally, the infant with CP exhibited higher MPF in the rubrospinal tract (Fig. 7), potentially reflecting greater reliance on this alternate motor pathway following CST injury. Similar mechanisms have been observed in other neurological conditions (Owen et al., 2017), including increased reticulospinal excitability in adults with CP(Smith and Gorassini, 2018). These findings support the potential of non-CST pathways, such as the rubrospinal and reticulospinal tracts, to compensate for CST damage, reinforcing the importance of validating these imaging and behavioral biomarkers in broader CP cohorts.

### 4.4 Differentiating early motor delay from CP

One infant in our cohort, who experienced gross motor delay at 18 months (Bayley >2 SD below the mean) but walked independently by age two, exhibited BabyOSCAR scores and CST imaging metrics (MPF, FA) within the 95% CI for typical development. This case indicates that early motor delay without structural CST abnormalities might reflect transient developmental differences rather than permanent injury. Using behavioral and imaging measures for early differentiation may improve prognostic accuracy and enable more targeted intervention strategies.

### 4.5 Limitations and future directions

The primary limitation of this study is its small sample size, which includes only one infant with cerebral palsy. While this case provides valuable preliminary insights, it limits generalizability. Only 5 children had repeat MRI scans, leaving the other children with a single-time-point MRI. Future studies should include larger cohorts with varied perinatal brain injuries to validate these findings and assess the clinical utility of MPF and g-ratio as early biomarkers for motor impairment.

Longitudinal follow-up is essential for linking early MRI findings with long-term motor and cognitive outcomes. Future studies should also explore the mechanisms underlying compensatory changes in the contralateral hemisphere and the rubrospinal tract, as well as the dissociation between MPF and FA metrics in the corpus callosum.

In conclusion, multimodal imaging, including advanced myelin mapping and diffusion-based fiber tract density, could enhance prognostic accuracy by providing insights into the timing, efficiency, and scope of motor circuit development. Identifying structural markers related to emerging SMC might strengthen the biological basis for early diagnosis and facilitate more targeted, timely interventions in infants with atypical motor development.

## Funding sources

CP was supported, in part, by the National Institutes of Health's National Center for Advancing Translational Sciences, Grant Number KL2TR001424.

## CRediT authorship contribution statement

**Alexander Drobyshevsky:** Writing – review & editing, Writing – original draft, Formal analysis, Conceptualization. **Vasiliy Yarnykh:** Software, Methodology. **Theresa Sukal Moulton:** Writing – review & editing, Methodology, Formal analysis, Data curation. **Andrea C. Pardo:** Writing – review & editing, Data curation. **Raye Ann deRegnier:** Writing – review & editing, Data curation. **Colleen Peyton:** Writing – review & editing, Project administration, Funding acquisition, Formal analysis, Data curation, Conceptualization.

## Data Availability

Data will be made available on request.
